# The world we want: focus on the most disadvantaged

**DOI:** 10.3402/gha.v6i0.20919

**Published:** 2013-04-10

**Authors:** Sarah Thomsen, Xu Biao, Hari Kusnanto, Dileep Mavalankar, Mats Målqvist, Nawi Ng, Vinod Diwan

**Affiliations:** 1 Karolinska Institutet, Solna, Sweden; 2 Fudan University, Shanghai, China; 3 University of Gadja Madah, Yogyakarta, Indonesia; 4 Indian Institute of Public Health, Kolkata, India; 5 Uppsala University, Uppsala, Sweden; 6 Umeå University, Umeå, Sweden; 7 Karolinska Institutet, Solna, Sweden

The global commitment to the Millennium Development Goal (MDG) process has resulted in significant, positive changes in health-related MDGs on the global and country levels since 1990 (1). However, while overall progress has been made, gaps in achievements between and within many countries have not decreased, with the poorest and most disadvantaged communities being the least likely to have benefitted. This is particularly the case in many emerging economies where the gap between the rich and poor, educated and uneducated, and minority and majority ethnic populations is actually increasing. For example, in India, where the Gross National Income in purchasing power parity in 2010 was $3,468, use of antenatal care services increased by 12% from 1996 to 2008, but only 0.1% among the poor (2). In Indonesia, infant mortality rates are on the decline in all regions of the country except for the Eastern regions where they remain high (3). In Vietnam, inequity in home deliveries between poor, rural Kinh (majority) and minority mothers has increased in the last 5 years during a period of rapid economic growth (4). In urban China, domestic rural-to-urban migrants account for a significant proportion of notified cases of infectious diseases such as tuberculosis (5), which is mainly associated with the low-income, poor living conditions, limited access to health care and vulnerability to poor health of this population, and their exclusion from benefits for local residents – such as health insurance (6).

An unintended focus on national MDG targets presented a disincentive to focus on equity by promoting ‘cherry picking’ (7). In effect, the focus on targets in achievement of the Millennium Development Goals has created an incentive for governments to implement utilitarian approaches to health as opposed to universalist ones, in the hopes of achieving ‘trickle-down’ effects (8). The result of this has been to create a greater disparity between those lifted ‘above the poverty line’ and those left behind (9).

The UN System Task Team has suggested a ‘single, high level goal’ with an ‘equity dimension’ as an outcome of the current global consultations for a post-2015 health goal (10). Universal health coverage, a product of a universalist approach to health, has been proposed as a worthy post-2015 health option. The establishment of health systems that are accessible and affordable to all is a worthy goal, but, we feel, not sufficient. Health interventions associated with MDGs 4, 5, and 6, for instance, are mainly applied through established health services to which only a segment of the population have easy access, usually the same fraction for all interventions (11). Therefore, the truly disadvantaged will not be reached through universal health coverage. A better alternative is to focus even more on disadvantaged populations while at the same time work towards a universal health system. The Marmot Review has referred to such policies as ‘proportionate universalism’ (12).

Arguments for focusing on disadvantaged populations specifically while building up a universal health system are based on human rights, and on the need for healthy populations to promote sustainable development (10, 13). Disadvantaged populations are *by definition* more likely than the general population to be at risk of experiencing the exact causes of morbidity and mortality that public health systems are designed to prevent. This is because of their greater exposure to social and structural determinants of ill-health such as low socio-economic position and lack of social capital (14). In addition to the moral issues around health inequities, pockets of high-risk populations also pose risks to the general population. The most obvious example is infectious diseases. The wealthy have a lower risk of disease given their living conditions and nutrition but also a higher frequency of immunization. Conversely, the poor have a higher risk of disease and often a low frequency of immunization. As these two populations often meet due to work or otherwise, the transmission may still take place between the populations, thus defeating the purpose of the public health intervention. Thus, full immunization of populations with higher risk is cost effective in a utilitarian health system.

Five papers previously published in *Global Health Action*, and now also printed in a compendium together with this Guest Editorial (numbered I-V), address this very issue of inequity and disadvantaged populations.

Thomsen et al. in ‘Bringing evidence to policy to achieve health-related MDGs for all: justification and design of the EPI-4 project in China, India, Indonesia, and Vietnam’ (15, I) report that to understand country-level MDG achievements it is useful to analyze their social and structural determinants. This analysis is not sufficient, however, to understand within-country inequities. Specialized analyses are required for this purpose, as is discussion and debate of the results with policymakers, which is the aim of the EPI-4 project. The purpose of this article is to set out the relevance and design of the ‘Evidence for Policy and Implementation project (EPI-4)’. EPI-4 aims to contribute to the reduction of inequities in the achievement of health-related MDGs in China, India, Indonesia, and Vietnam through the promotion of research-informed policymaking.

Sanneving et al. in ‘Inequity in India. The case of maternal and reproductive health’ (16, II) report five main structural determinants emerged from the analysis as important in understanding equity in India: economic status, gender, education, social status (scheduled caste or tribe), and age (adolescents). The authors conclude that in India, economic status, gender, and social status are all closely interrelated when influencing use of and access to maternal and reproductive health care.

Saxena et al. in ‘Inequity in maternal health care service utilization in Gujarat: analyses of district level household survey data’ (17, III) report inequities in maternal health care utilization in Gujarat. Structural determinants like caste group, wealth, and education were all significantly associated with access to the minimum three antenatal care visits, institutional deliveries, and use of any modern method of contraceptive. There is a significant relationship between being poor and access to less utilization of ante-natal care services independent of caste category or residence. The report concludes that poverty is the most important determinant of non-use of maternal health services in Gujarat. In addition, social position (i.e. caste) has a strong independent effect on maternal health service use. More focused and targeted efforts towards these disadvantaged groups need to be taken at policy level in order to achieve targets and goals laid out as per the MDGs.

Yuan et al. in ‘Disadvantaged populations in maternal health in China – Who and why?’ (18, IV) report that in China, differences in maternal health service utilization and maternal mortality ratio among different income groups and among regions with different socio-economic development still exist, although these differences are narrowing, and conclude that inequity in maternal health continues to be an issue worthy of greater programmatic and monitoring efforts in China.

Målqvist et al. ‘Ethnic minority health in Vietnam: a review exposing horizontal inequity’ (19, V) report five main areas (health-care-seeking and utilization, maternal and child health, nutrition, infectious diseases, and oral health and hygiene) where equity in health is a pressing concern and reaching disadvantaged populations is necessary to close the inequity gap. Research evidence also offers explanations derived from both external and internal group dynamics to this inequity.

*Sarah Thomsen* Karolinska Institutet, Solna Sweden *Xu Biao* Fudan University, Shanghai China *Hari Kusnanto* University of Gadja Madah, Yogyakarta Indonesia *Dileep Mavalankar* Indian Institute of Public Health, Kolkata India *Mats Målqvist* Uppsala University, Uppsala Sweden *Nawi Ng* Umeå University, Umeå Sweden *Vinod Diwan* Karolinska Institutet, Solna Sweden
